# NMDA 2A receptors in parvalbumin cells mediate sex-specific rapid ketamine response on cortical activity

**DOI:** 10.1038/s41380-018-0341-9

**Published:** 2019-01-29

**Authors:** Nathalie Picard, Anne E. Takesian, Michela Fagiolini, Takao K. Hensch

**Affiliations:** 1 FM Kirby Neurobiology Center, Department of Neurology, Boston Children’s Hospital, Harvard Medical School, 300 Longwood Avenue, Boston, MA 02115 USA; 2 Center for Brain Science, Department of Molecular Cellular Biology, Harvard University, 52 Oxford Street, Cambridge, MA 02138 USA

**Keywords:** Neuroscience, Depression

## Abstract

Ketamine has emerged as a widespread treatment for a variety of psychiatric disorders when used at sub-anesthetic doses, but the neural mechanisms underlying its acute action remain unclear. Here, we identified NMDA receptors containing the 2A subunit (GluN2A) on parvalbumin (PV)-expressing inhibitory interneurons as a pivotal target of low-dose ketamine. Genetically deleting GluN2A receptors globally or selectively from PV interneurons abolished the rapid enhancement of visual cortical responses and gamma-band oscillations by ketamine. Moreover, during the follicular phase of the estrous cycle in female mice, the ketamine response was transiently attenuated along with a concomitant decrease of *grin2A* mRNA expression within PV interneurons. Thus, GluN2A receptors on PV interneurons mediate the immediate actions of low-dose ketamine treatment, and fluctuations in receptor expression across the estrous cycle may underlie sex-differences in drug efficacy.

## Introduction

For 20 years, ketamine has received considerable attention for its preclinical and clinical applications when used at sub-anesthetic doses. Low-dose ketamine in adulthood has been shown to produce antidepressant effects [1–5] and to relieve suicidal ideation [6] or post-traumatic disorders [7]. Recently, chronic administration of ketamine has been described as a promising potential treatment in neurodevelopmental models of Rett (RTT) syndrome [8]. While ketamine is generally known as a non-competitive NMDA receptor antagonist at high doses, its broader mode of action remains to be elucidated.

Notably, it is unclear how low-dose ketamine triggers its rapid action and if similar processes act in males vs females [9, 10]. Indeed, whether NMDA receptors primarily mediate ketamine effects is now highly debated as recent studies point to other systems such as dopaminergic [11], serotonergic [12], sigma [13], opioid [14], GABA [15], or AMPA receptors [10] as potentially relevant. On the other hand, a prevalent body of clinical and preclinical evidence implicates a preferential blockade of NMDA receptors on parvalbumin-expressing (PV) interneurons as a central mechanism of classical ketamine action, such as increased cortical activity and γ-band oscillations (GBO) in the 30–80 Hz range [16–20].

NMDA receptors are heteromeric complexes composed of at least one GluN1 subunit and one or more GluN2A-D subunits, which define the functional properties of the receptor [21]. Importantly, this subunit composition dynamically changes over the development in both pyramidal and PV-cells with a cell-specific time course [22–24]. Although NMDA receptors mainly contain GluN2A subunits in adulthood [21], several studies have suggested that the less prevalent GluN2C/2D-containing NMDARs might have an important role in mediating ketamine action [25, 26] and the role of the GluN2A subunit in the rapid action of ketamine has never been tested.

Here, we directly examined the hypothesis that GluN2A subunits in PV-cells mediate the rapid actions of acutely injected, low-dose ketamine (8 mg/kg, i.p.). We used gene-targeting in mice to delete GluN2A globally or specifically from PV-cells, and demonstrate the absence of ketamine-induced changes in pyramidal cell activity and GBO in the primary visual cortex (V1). We further compared the ketamine response in male mice to that of females during follicular (Estrus/Proestrus) and luteal (Metestrus/Diestrus) phases of the estrous cycle, revealing that females during the follicular phase do not display the typical response to ketamine. This transient, natural loss of ketamine sensitivity in females was correlated with a shift toward lower gene expression of *grin2a* (coding for GluN2A) among PV-cells as compared to the luteal stage. Our findings carry broad implications for the therapeutic use of other more specific NMDA receptor antagonists given the wide gender-based differences in psychiatric illnesses [27–29].

## Materials and methods

All procedures were approved by the Institutional Animal Care and Use Committee (IACUC) at Boston Children’s Hospital and performed in adult mice aged between P60 and P90 except where stated otherwise.

### Animals

Mice were maintained on a C57BL/6J background kept on a 12-h light/dark cycle and provided with food and water ad libitum. Wild-type males were pooled across the different lines as no difference was observed in their response to ketamine (Supp Fig. 1b and c). We generated conditional PV-Cre/GluN2A^f/f^ mice by crossing PV-IRES-Cre mice (JAX 008069) with mice carrying “floxed” *GluN2A* alleles, originally provided by Dr. K. Sakimura (Niigata University) [30]. Constitutive GluN2A^−/−^ mice were back-crossed >11 generations onto C57Bl/6J from original breeding pairs provided by Dr. M. Mishina (University of Tokyo) [31]. PV-GFP transgenic mouse breeders were originally provided by Dr. H. Monyer (Heidelberg University) [32]. For each group, mice were taken across different litters.

### Identification of the mouse estrous cycle

We performed vaginal cytology as defined previously [33]. Stages of the estrous cycle were determined by observing the presence of leukocytes, cornified epithelial cells, and nucleated epithelial cells in the fluid. We followed the estrous cycle for a few days before the experiment and confirmed the stage on the day of the experiment.

### In vivo single-unit recordings

Mice were anesthetized under Nembutal (50 mg/kg, i.p.)/chlorprothixene (0.025 mg/kg, i.p.) using standard techniques [34]. Additionally, atropine (0.3 mg/kg) and dexamethasone (2 mg/kg) were administered subcutaneously to reduce secretions and edema, respectively. Cortical activity in response to visual stimulation in the binocular zone of V1 was recorded using multichannel probes (A1x16–3 mm 50_177, Neuronexus Technologies).

#### Gamma power analysis

Laminar location was evaluated with contrast-reversing (0.5 or 1 Hz) square checkerboard patterns (0.04 cpd, 25–50 repeats). For local field potential (LFP) recordings, the extracellular signal was filtered (1–300 Hz) and sampled at 1.5 kHz. Current source density (CSD) was computed from the average LFP as previously described [34] using the CSD plotter toolbox. The neural signal was averaged for all electrodes in layer 2/3 and further filtered (4–100 Hz). Power spectra were generated by fast Fourier transform on the average response across trials (1 s), starting 500 ms after horizontal sine wave grating onset, and analyzed (Chronux).

### In vitro whole-cell recordings

Acute coronal slices of binocular V1 (300 µm) were obtained (Leica Microsystems, VT1200S) from PV-GFP transgenic mice. Whole-cell recordings were made from PV-cells identified by fluorescence. Isolated NMDA receptor-mediated excitatory post-synaptic potentials (EPSC_NMDA_) were evoked by pulses of electrical stimulation delivered to cortical layer 4. A stimulus intensity was chosen that elicited a maximal EPSC_NMDA_ (30–100 μA), which was further isolated using a cocktail of drugs (bicuculline, 10 µM, Sigma; SCH-50911, 10 µM, Tocris Bioscience; CNQX, 20 µM, Tocris Bioscience; atropine, 1 µM, Sigma). The effects of ketamine (10 µM; Hospira Inc.) on EPSC_NMDA_ amplitudes were evaluated 15 min after bath application. The NMDA receptor-mediated component was verified using NMDA receptor antagonists (CPP, 20 µm; AP-5, 50 µM; Tocris Bioscience). Custom-designed IGOR (WaveMetrics) programs were used for data acquisition and analysis.

### RNA in situ hybridization

Isoflurane-anesthetized mice were decapitated and their brains removed, embedded in OCT compound (Tissue-Tek) and frozen on dry ice. Coronal brain slices (25 µm) through V1 were cut on a cryostat (Leica CM 1900), adhered to SuperFrost Plus slides (VWR), and immediately refrozen on dry ice. Sections were fixed in 4% paraformaldehyde and dehydrated in increasing concentrations of ethanol. Hybridization and amplification steps were then performed using the RNAscope Multiplex Assay protocol (Advanced Cell Diagnostics Inc., Hayward, CA) and the following fluorophore-conjugated RNAscope probes: Mm-*Grin2a*-C1 (Cat No. 481831) and Mm-*PVALB*-C2 (Cat No. 461691). Confocal images were acquired on a Zeiss LSM 710 microscope using a 63×, 1.4 NA oil immersion PlanAPO objective (1.1× zoom) and quantified with Fiji. ROIs to delineate the cytoplasm of *PVALB*-cells were drawn manually blind to groups, and integrated density (ROI area × mean intensity) was computed for each group.

### Statistical analysis

All data are presented as median ± CI unless specified. Datasets were first analyzed using the Kolmogorov–Smirnov test for normality. Because they did not display normal distribution, non-parametric tests were used to assess differences. In each group, the effect of ketamine over time was tested using Friedman test with Dunn’s multiple comparison. Differences between groups were analyzed using Kruskal–Wallis with Dunn’s multiple comparison test. Two-way RM-ANOVA (matching across row) with Bonferroni’s post-test was used for power spectra analysis. A paired *t* test was used to evaluate in vitro effects of ketamine on EPSC_NMDA._ Statistical analyses were performed using GraphPad version 5.0 (Prism) or JMP (SAS Institute) statistical software. In all figures, *p < 0.05, **p < 0.01, ***p < 0.001, and ****p < 0.0001.

## Results

### NMDA-GluN2A receptor mediates rapid enhancement of cortical activity by ketamine

**Fig. 1:**
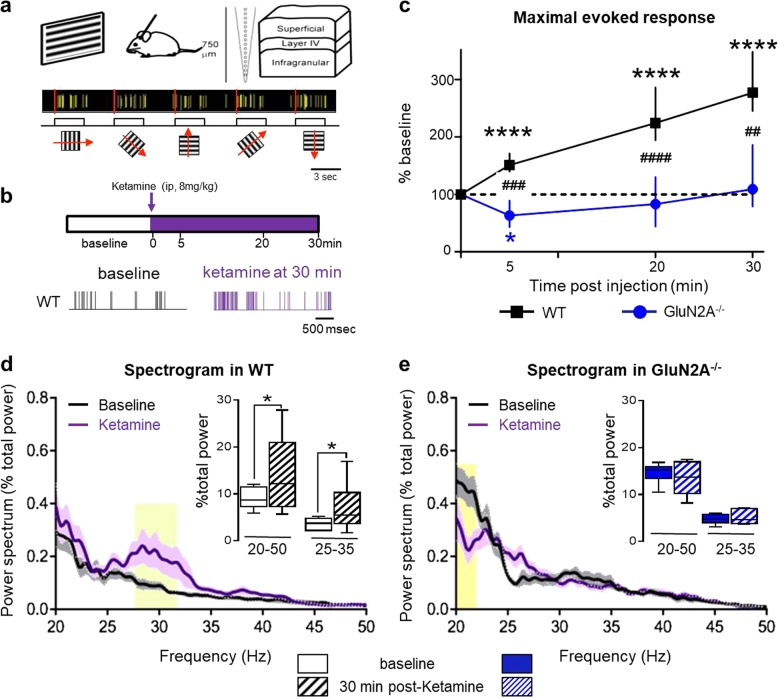
GluN2A receptors mediate the rapid actions of low-dose ketamine. **a** Evoked responses from primary visual cortex in vivo using a multisite silicon probe through the depth of cortex. Pyramidal-cell activity was recorded in response to drifting gratings of different orientation (30° spacing; 3–10 repeats) to determine the maximal evoked response for each cell. **b** Pyramidal-cell activity was recorded before/after one injection of low-dose ketamine (8 mg/kg, i.p.). Rapid changes in activity were evaluated over 30 min post-injection (top). Representative example of cell activity before and after 30 min of ketamine in wildtype controls (WT; bottom). **c** Ketamine-induced changes of maximal evoked response in WT (black filled square, *n* = 171 cells; 13 males) and GluN2A^−/−^ mice (blue filled circle, *n* = 54 cells; 6 males) (median ± 95% CI. Asterisk refers to Friedman test with Dunn’s multiple comparison vs baseline. Hash refers to Kruskal–Wallis with Dunn’s multiple comparison WT vs GluN2A^−/−^ mice). **d** Spectrogram comparison from WT at baseline (black trace) and 30 min post-ketamine (purple trace). Solid lines, mean of all mice; shaded areas, sem. Spectrogram calculated as a percentage of total power measured between 4 and 100 Hz (*n* = 10 mice). *p*-Value of two-way RM ANOVA: yellow shading, regions with statistically significant effect (Sidak’s multiple comparison *p* < 0.05). Inset, quantification of γ-oscillations between 20–50 Hz and 25–35 Hz (asterisk refers to Wilcoxon paired test baseline vs 30 min). **e** Spectrogram comparison from GluN2A^−/−^ mice at baseline and 30 min post-ketamine (*n* = 6 mice). Inset, quantification of γ-oscillations between 20–50 Hz and 25–35 Hz

Visually-evoked responses to drifting gratings at different orientations were recorded in anesthetized adult male mice (P60–80) using a multisite silicon probe through the depth of V1 (Fig. 1a). The maximal evoked response was determined for each regular spiking (putative pyramidal) cell before and 30 min after ketamine injection (8 mg/kg, i.p.; Fig. 1b). Importantly, we analyzed our data at an early time point (5–30 min post-injection), when ketamine concentration peaks in the brain [8, 10].

In wildtype (WT) mice, ketamine induced a rapid and sustained increase of maximal evoked firing, that was immediately significant and persisted throughout the recording (Friedman test *p* < 0.0001, Dunn’s multiple comparison T5 vs T0, *p* < 0.0001, T20–30 vs T0 *p* < 0.0001; Fig. 1c). Strikingly, genetic ablation of GluN2A abolished the ketamine effect: in GluN2A^−/−^ mice, the maximal evoked response first decreased at 5 min post-injection (Friedman test *p* = 0.0011, T0 vs T5, *p* = 0.048) then recovered to baseline levels (T0 vs T20 *p* = 0.64, vs T30, *p* = 0.49, Fig. 1c). The response to ketamine was significantly different from WT at all time points (Kruskal–Wallis test *p* < 0.0001; Dunn’s multiple comparison at: 5 min *p* = 0.0004, 20 min *p* < 0.0001, and 30 min *p* = 0.0041).

Ketamine at low doses is well known to modulate neuronal oscillatory activity in humans and rodents [17, 18, 35]. Oscillatory activity at γ-frequency (30–80 Hz) is implicated in information processing, memory, and sensory perception and used as an index of network activity and cognitive performance [36]. In particular, ketamine alters these GBO that are generated by PV-cells [37]. To evaluate a role for the GluN2A receptor in the ketamine response at the network level, we therefore measured visually-driven GBO in superficial cortical layers. In mouse V1, visual stimulation triggers GBO mainly between 20 and 50 Hz that are not time-locked to the stimulus onset [38]. Visually-driven GBO were measured before and 30 min post-ketamine injection for 1 s starting 500 ms after horizontal grating stimulus onset.

In WT, spectrogram power analysis revealed an overall increase of GBO between 20 and 50 Hz (row matched two-way ANOVA, *p* < 0.0001), including an especially prominent enhancement from 25 to 35 Hz (yellow shading; Sidak’s multiple comparison, *p* < 0.05, Fig. 1d). Quantification of GBO power confirmed a significant increase 30 min post-ketamine injection (Fig. 1d, asterisk: Wilcoxon test *p* = 0.03). In GluN2A^−/−^ mice, this ketamine-induced increase of GBO was absent (Fig. 1e, *p* = 0.34). Spectrogram analysis instead revealed a decrease in the low range of GBO (between 20 and 22 Hz, Fig. 1e). Taken together, these results reveal that GluN2A receptors contribute significantly to the classical acute ketamine response.

### NMDA-GluN2A receptors on PV-cells underlie rapid ketamine action

**Fig. 2:**
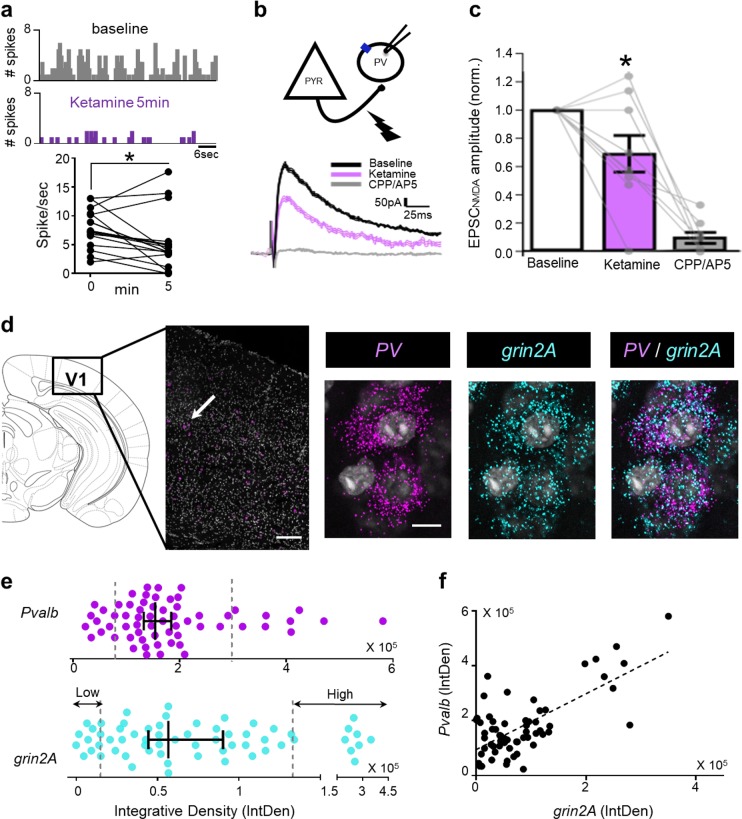
Rapid ketamine action in fast-spiking PV-cells. **a** Representative raster plots of fast-spiking cells in V1 recorded in vivo at baseline and 5 min post-ketamine injection (top). Firing rate changes in these putative inhibitory cells recorded in response to visual gratings (*n* = 17, Wilcoxon matched paired test, *p* = 0.038) (bottom). **b** Representative NMDA receptor-mediated excitatory post-synaptic currents (EPSC_NMDA_) recorded from PV-cells in vitro in WT males at baseline (black traces), 15 min after bath application of ketamine (10 µM, purple line) then CPP/AP5 (gray trace). **c** Quantification of EPSC_NMDA_ amplitude normalized to baseline (mean ± sem, *n* = 9 cells, 6 mice; matched paired test, *F* = 5.85, *p* = 0.042). **d** Representative in situ hybridization images from WT mice depicting co-localization of *grin2A* and interneuron marker (*Pvalb*) in visual cortex. **e** Integrated density (IntDen) of *Pvalb* and *grin2A* in PV-positive cells (median ± 95% CI, *n* = 62 cells). **f** Positive correlation between *Pvalb* and *grin2A* expression (Spearman *p* = 0.0005)

While it has been proposed that PV interneurons preferentially mediate the low-dose ketamine response [16, 39], little is known about how these cells are acutely modulated by the drug. Identified on the basis of their average waveform properties, we isolated narrow-spiking cells (putative inhibitory PV interneurons) from regular-spiking, presumptive pyramidal cells (Supp Fig. 1a). Unlike the latter, putative PV-cell mean activity was reduced in WT mice immediately after ketamine injection in comparison to their baseline firing rates (Fig. 2a and Supp Fig. 1d,e).

To further understand the role of NMDA receptors on PV-cells in the rapid ketamine action, we recorded NMDA receptor-mediated excitatory postsynaptic currents (EPSC_NMDA_) from PV-cells in brain slices of V1 in vitro (Fig. 2b). EPSC_NMDA_ were isolated using a cocktail of drugs, and evoked by extracellular stimulation (200 μs, 30–100 μA). Bath-applied ketamine (10 μM) significantly reduced EPSC_NMDA_ amplitude (69 ± 13% of baseline, *n* = 9 cells, matched pairs test, *p* = 0.042) with some variability across PV-cells. Whereas the majority showed a pronounced decrease of EPSC_NMDA_ amplitude, a few cells showed little effect of ketamine. The NMDA receptor-mediated component was then verified using NMDA receptor antagonists, CPP and AP5, which abolished the current (9.5 ± 4.1% of baseline, *n* = 8 cells; Fig. 2b, c).

Mature NMDA currents recorded in cortical PV cells are primarily mediated by 2A subunits [24, 40]. Using in situ hybridization in V1, we quantified co-expression of mRNAs encoding *grin2A* and *Pvalb* (Fig. 2d) and confirmed that GluN2A was heterogeneously expressed among PV-cells. Cells exhibiting high expression (IntDensity > 85% percentile) and those showing low expression (IntDensity < 15% percentile) were identified (Fig. 2e). Notably, Spearman correlation revealed a significant relationship between *Pvalb* and *grin2A* expression (*R* = 0.427, *p* = 0.0005, Fig. 2f). Interestingly, heterogeneity was present in all layers (Supp Fig. 2).

**Fig. 3:**
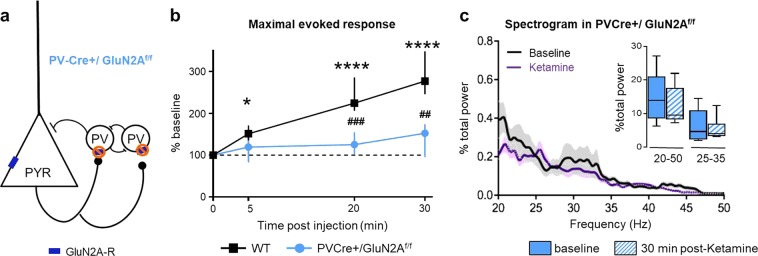
GluN2A receptors on PV-cells mediate rapid ketamine action. **a** Schematic of PV-Cre^+^/GluN2A^f/f^ mouse construction. **b** Ketamine-induced changes of maximal evoked response were abolished in PV-Cre^+^/GluN2A^f/f^ males (blue filled circle, *n* = 72, 6 mice) (% of pre-ketamine level, median ± 95% CI; asterisk refers to Friedman test with Dunn’s multiple comparison vs baseline; hash refers to Kruskal–Wallis with Dunn’s multiple comparison WT vs mutant). **c** Spectrogram comparison from WT at baseline (black trace) and 30 min post-ketamine (purple trace). Solid lines, mean of all mice; shaded areas, sem. Spectrogram calculated as a percentage of total power measured between 4 and 100 Hz (*n* = 6 mice). *p*-Value of two-way row matched ANOVA. Inset, quantification of γ-oscillations between 20–50 Hz and 25–35 Hz

To directly determine whether the GluN2A receptor on PV-cells is necessary for rapid ketamine effects on cortical activity, we selectively deleted these subunits using Cre-recombinase technology (PV-Cre^+^/GluN2A^f/f^ mice; Fig. 3a). In contrast to WT animals, ketamine did not significantly increase maximal evoked responses in PV-Cre^+^/GluN2A^f/f^ mice (Fig. 3b; Friedman test *p* = 0.1467). As a result, ketamine action was significantly greater in WT than in PV-Cre^+^/GluN2A^f/f^ mice at 20 and 30 min after injection (Kruskal–Wallis *p* < 0.0001 with Dunn’s multiple comparison test, *p* < 0.001, Fig. 3b). Deleting GluN2A exclusively from PV-cells also abolished the ketamine-induced increase of GBO (Fig. 3c).

Together, our results reveal a major role for NMDA receptors—in particular those containing the GluN2A subunit in PV-cells—in the rapid actions of ketamine on cortical neuronal activity. Importantly, deleting GluN2A from PV-cells alone mimics the effect of total GluN2A deletion.

### Ketamine action is disrupted in females during estrous

**Fig. 4:**
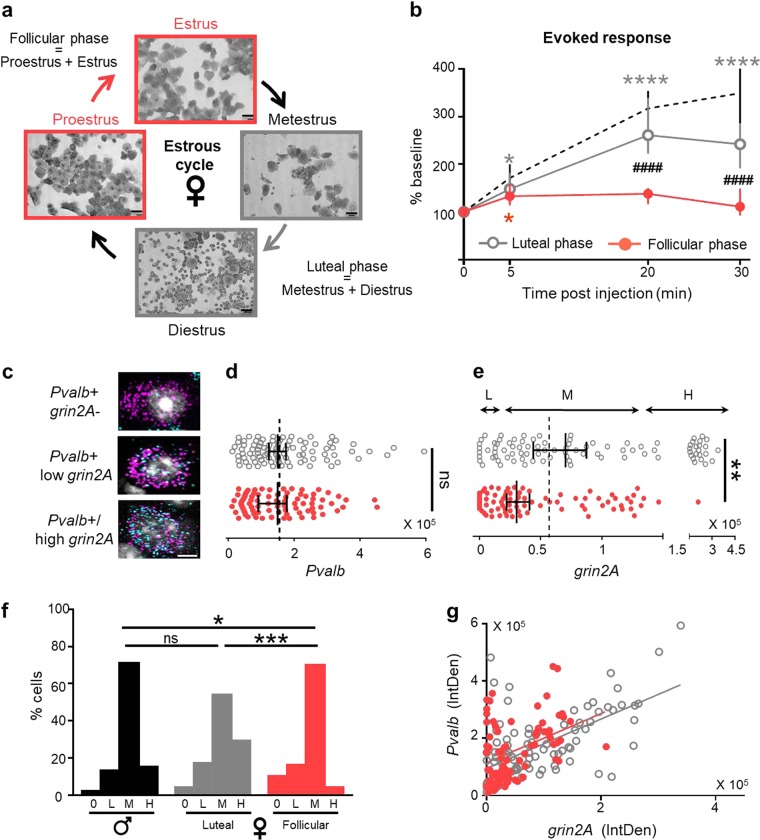
Attenuated female response to ketamine during the follicular phase. **a** Representative vaginal smears during Estrus, Metestrus, Diestrus, and Proestrus stages of the estrous cycle. **b** Ketamine-induced changes of maximal evoked response in C57Bl/6J females during the follicular phase (Estrus/Proestrus, red filled circles, *n* = 87 cells; 5 mice) and luteal phase (Metestrus/Diestrus, gray circles, *n* = 82 cells; 6 mice) (median ± 95% CI; asterisk refers to Friedman test with Dunn’s multiple comparison vs baseline; hash refers to Kruskal–Wallis with Dunn’s multiple comparison follicular vs luteal). Dotted line, C57Bl/6J male values for comparison (*n* = 67 cells/4 mice). **c** Representative in situ hybridization images of female mouse V1 showing different categories of *Pvalb*-positive cells. **d** Integrated density of *Pvalb* mRNA in luteal and follicular stages (median ± 95% CI, *n* = 81 and 90 cells; Kruskal–Wallis test, not significant). **e** Integrated density (IntDen) of *grin2A* mRNA in luteal and follicular stages (Mann–Whitney, *p* = 0.0016). Dotted line, median value in males. **f** Proportion of *Pvalb*-positive cells without *grin2A* (0), low level (L: IntDen < 15% percentiles in males), medium (M) or high level of *grin2A* expression (H: IntDen > 85% percentiles in males). Males (black) and females during luteal (gray) and follicular (red) phases (asterisk refers to *χ*^2^-test). **g** Positive correlation between *Pvalb* and *grin2A* expression in females

Our results suggest that modulating NMDA receptor activity could influence the rapid ketamine response. Interestingly, NMDA receptors are altered by gonadal hormones. Estradiol increases NMDA receptor density in rat hippocampus [41–45], modulates its activity/sensitivity [45, 46], and also modifies its phosphorylation status [47]. Behavioral studies have suggested that female rodents may be differentially sensitive to NMDA receptor antagonists than males [48–51]. Thus, we evaluated the rapid action of ketamine in WT females according to their estrous cycle as determined by vaginal smears (Fig. 4a).

Females were classified in two groups: (1) follicular phase (Estrus + Proestrus) when estradiol concentration is low, and (2) luteal phase (Metestrus + Diestrus) when estradiol concentration is high [33, 52]. In luteal females, the ketamine-induced increase of evoked response was similar to that observed in males (Supp Fig. 3a). In contrast, the administration of ketamine to females during the follicular stage elicited only weak, transient effects on neuronal activity. The evoked response increased slightly at 5 min (127 ± 8% of baseline, Friedman *p* = 0.0319) but was no different from baseline at 20 and 30 min. As a result, the response to ketamine was significantly different from both males (Supp Fig. 4c) and females during the luteal phase (Fig. 4b; KW *p* < 0.0001, Dunn’s at T20 and 30 *p* < 0.0001).

To identify a mechanism underlying this attenuated, estrus state-dependent response to ketamine in females, we compared *Pvalb* and *grin2a* mRNA expression between males and females during luteal or follicular phases. No differences were observed between groups in *Pvalb* expression (Fig. 4d). However, in female mice during the follicular phase, the overall expression of *grin2a* was significantly lower in comparison to males and luteal females (Fig. 4e). In particular, the distribution of cells with zero, low, and high expression was significantly different in follicular females (Fig. 4f; *χ*^2^-test, male vs follicular *p* = 0.01, male vs luteal *p* = 0.68, luteal vs follicular *p* = 0.0006). Instead, the correlated expression between *Pvalb* and *grin2A* was preserved in both groups of females (Fig. 4g; follicular *R* = 0.391, *p* = 0.0003; luteal *R* = 0.605, *p* < 0.0001).

## Discussion

Our findings clarify how low-dose ketamine triggers its rapid action within the neocortex. Acute sub-anesthetic ketamine administration in healthy volunteers induces schizophrenia-like symptoms and neurocognitive deficits while impacting thalamo-cortical processing distinct from the disease state [53]. At clinically relevant low-dose, we found that cortical response enhancement is borne by 2A subunit-containing NMDA receptors localized to a subset of PV-positive, fast-spiking interneurons. Moreover, we found that ketamine sensitivity was transiently absent in females during the follicular phase of their estrous cycle, associated with a decreased percentage of PV-cells expressing high *grin2a* levels.

Ketamine has been shown to have rapid and long-lasting, beneficial effects as a potential treatment for depression, suicidal ideation, or post-traumatic stress disorder. Yet, the mechanism underlying such actions remains under intensive debate. Recently, it was reported to act through NMDA receptors on glutamatergic neurons within the lateral habenula leading to disinhibition of reward center pathways to relieve depression [54]. We verified a further role for the GluN2A subunit in such antidepressant effects at the low doses of ketamine used here. After saline injection, GluN2A^−/−^ mice displayed a reduced immobility time in the forced swim test (Supp Fig. 4a), which occluded further antidepressant like effects of ketamine (Supp Fig. 4b,c). Moreover, ketamine-induced decrease in Akt and eEF2 phosphorylation in the frontal cortex was attenuated in GluN2A^−/−^ mice (Supp Fig. 4d–f). A deficit in neuronal activity in the prefrontal cortex has been suggested in both clinically depressed humans and mouse models, while optogenetic stimulation therein generates an antidepressant like effect in mice [55].

Our results are consistent with a disinhibition of cortical excitatory activity due to a greater NMDA sensitivity of inhibitory circuits [16, 56–60]. As in the anesthetized mouse V1 here, acute ketamine exposure in adulthood increases excitatory transmission in frontal and anterior cingulate cortex across species [61], as well as evoked pyramidal cell activity and GBO in awake rodents [16–19]. Moreover, the effects described here mirror those observed in healthy human subjects after ketamine injection [35, 62, 63], implicating shared mechanisms relevant for translation to humans. Interestingly, disinhibition of somatostatin-positive interneurons has recently been suggested to mediate the long-lasting antidepressant actions of selective serotonin reuptake inhibitors (SSRI), indicating a distinct inhibitory circuit logic may mediate rapid (PV-cells) or slow antidepressant effects [64, 65].

The subunit composition of NMDA receptors in PV-cells differs from neighboring pyramidal neurons, with GluN2A and GluN2C subunits being highly expressed [66, 67]. At physiological Mg2+ concentrations, NMDA receptor antagonists such as ketamine or memantine have little impact on reconstituted GluN2A- or GluN2B-containing receptors in vitro, while their blockade of GluN2C- or GluN2D-containing receptors is largely preserved [25]. Yet, deletion of GluN2B from pyramidal cells in the cortex and hippocampus may mimic and occlude ketamine actions on depression-like behavior, excitatory synaptic transmission, mTOR activation, and synaptic protein synthesis in response to six-fold higher drug doses (50 mg/kg) than used here [68]. Our findings reveal a pivotal role for GluN2A receptors in PV-cells underlying clinical, low-dose (8 mg/kg) ketamine action in the intact brain. This may reflect an enhanced sensitivity of GluN2A currents in these fast-spiking cells in vivo, which typically exhibit depolarized membrane potentials and high spontaneous input [69], as well as elevated redox regulation [70] known to rapidly and reversibly potentiate currents through NMDA receptors composed of GluN1:GluN2A subunits [71].

The PV circuit mechanism may also help to explain striking sex-differences in drug efficacy. We found that ketamine does not increase cortical responses in females during the follicular phase of the estrous cycle. The loss of sensitivity was associated with a population shift toward PV-cells transiently expressing less *grin2A* mRNA. Instead, for luteal females the response to ketamine was similar to that of males. Such transcriptional regulation of NMDA receptor mRNA during the estrous cycle was made evident only by our targeted examination of changes specifically within PV-cells. These modifications complement previous results showing that estradiol, which peaks during the luteal phase, increases NMDA receptor density more broadly [41–45]. Thus, fluctuations in estradiol might mediate the variable sensitivity to ketamine and other NMDA receptor antagonists in females [50].

Remarkably, just a subtle change in *grin2a* expression during estrus was associated with a complete loss of acute ketamine action on cortical activity, similar to the full GluN2A deletion in males. While fine-tuning of GluN2A levels in a subset of PV-cells may explain this effect, it is also possible that additional post-translational modifications of the NMDA receptor are taking place during the follicular stage. Estrogen modulation of NMDA receptor activity and sensitivity involves extranuclear estrogen receptors (ER) [45, 47, 72], including potential activation of several signaling pathways such as ERK and Akt which phosphorylate NMDA receptor subunits [47]. Of particular interest is ER-α which is expressed more during non-estrus phases [73] and could therefore be responsible for increased NMDA receptor function and greater ketamine efficacy. It is also possible that the oxidative profile of free radical oxygen species generation/clearance is highly dynamic and region-specific in the female brain [74, 75].

Our findings bear directly upon discrepancies in the literature whether ketamine has greater, lesser, or no different effect in men and women. The day of injection might greatly influence the acute benefits of ketamine and differences in treatment timing might explain the great variability (anywhere from 40 to 80%) across clinical trials, including a recent report of only 43% of women showing improvement after ketamine infusion [76]. Increased understanding of these mechanisms with regard to sex differences will help develop tailored therapies and identify new risk and/or protective factors for psychiatric disorders.

## Supplementary information

Supplemental material
