# Supplementary material for: NMDA 2A receptors in parvalbumin cells mediate sex-specific rapid ketamine response on cortical activity
Source: Mol Psychiatry. 2019 Jan 29;24(6):828–38. doi: 10.1038/s41380-018-0341-9 (PMC6756203; doi:10.1038/s41380-018-0341-9)
Supplement: Supplementary file 1 — Supplemental material [file 41380_2018_341_MOESM1_ESM.docx]

**NMDA 2A receptors in parvalbumin cells mediate sex-specific rapid ketamine response on cortical activity**

Nathalie Picard^1^, Anne E Takesian^1^, Michela Fagiolini^1*^, Takao K Hensch^1,2*^

**Supplementary Information**

**Supp Figure 1: Classification of electrophysiological responses *in vivo.***

**Supp Figure 2: Layer specificity of *grin2A* and *Pvalb* colocalization in WT males.**

**Supp Figure 3: Comparison of ketamine effect in females and males.**

**Supp Figure 4: Absence of ketamine induced anti-depressant effect in GluN2A^-/-^ mice.**

**Materials and Methods**


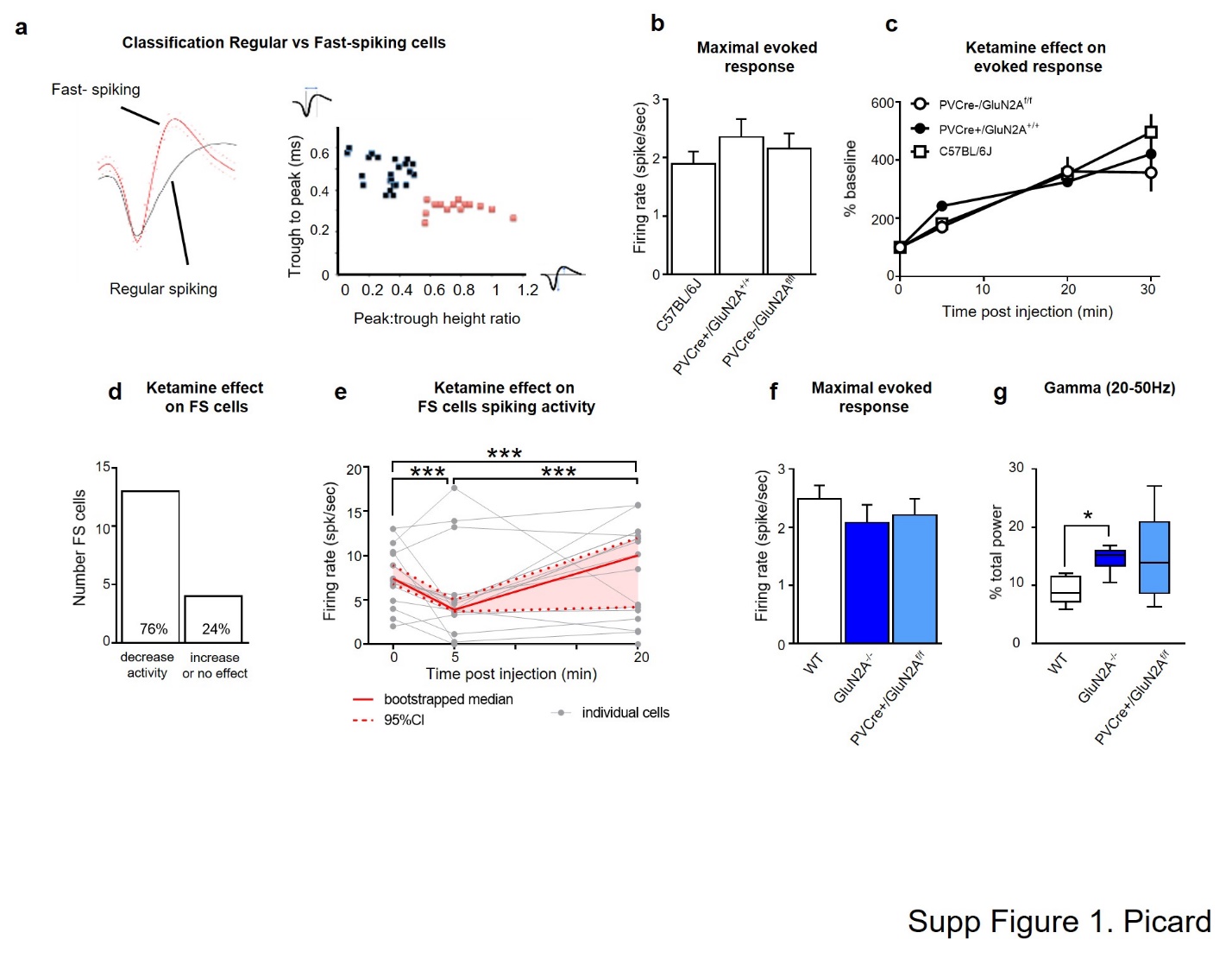


**Supp Figure 1**: **Classification of electrophysiological responses *in vivo.***

**a**) Left, Representative spike waveforms from broad- (black; regular-spiking) and narrow-spiking putative inhibitory cells (red; fast-spiking). Right, Regular- and fast-spiking cells were classified based on the width of the spike from trough to peak and the ratio of the amplitude of the spike trough to the amplitude of the spike peak. **b**) Comparison of evoked firing rates at baseline within three control groups later pooled in WT group for comparison to experimental group; C57Bl/6J (n = 67 cells / 4 mice), PV^+^/GluN2A^f/f^ (n = 62 cells / 5mice) and PVCre^-^/GluN2A^+/+^ (n = 42 cells / 4 mice) (Kruskal-Wallis with multiple comparison p= 0.65). **c**) Comparison of ketamine effect on evoked response in the 3 control groups (2-way ANOVA: source of variation: time p<0.0001, genotype p=0.533, interaction p=0.341). **d**) Proportion of fast-spiking (FS) cells that decreased firing rate 5 min after ketamine injection in WT males (n = 17 cells). **e)** Firing rate of FS cells following ketamine injection. Grey line, individual cells; red line, boot-strapped median ± 95%CI. **f)** Maximal evoked response in WT, GluN2A^-/-^ and PVCre^+^/GluN2A^f/f^ at baseline (Kruskal-Wallis p = 0.597) **g)** Gamma power in WT, GluN2A^-/-^ and PVCre^+^/GluN2A^f/f^ at baseline (Kruskal-Wallis p = 0.02; multiple comparison WT vs GluN2A^-/-^ p = 0.024; WT vs PVCre^+^/GluN2A^f/f^ p = 0.09).

**
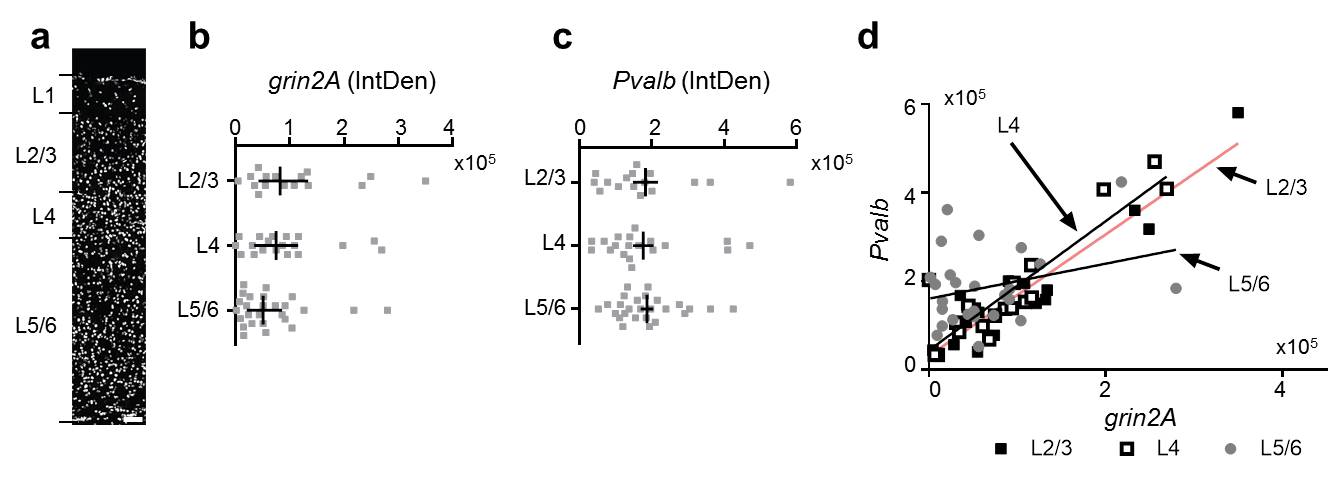
**

**Supp Figure 2**: **Layer specificity of *grin2A* and *Pvalb* colocalization in WT males.**

**a**) Sample DAPI staining to identify different layers in C57Bl/6J mouse visual cortex (scale, 50μm). **b**) *grin2A* mRNA expression quantified by Integrated Density (IntDen), the sum intensity of all pixel values within the cell. **c)** *Pvalb* expression IntDen. **d**) Correlation between *grin2A* and *Pvalb* expression by layer (n = 16 (L2/3), 19 (L4), 26 (L5/6) cells, respectively). Linear regression lines between *grin2A* and *Pvalb* IntDen by layer.

**
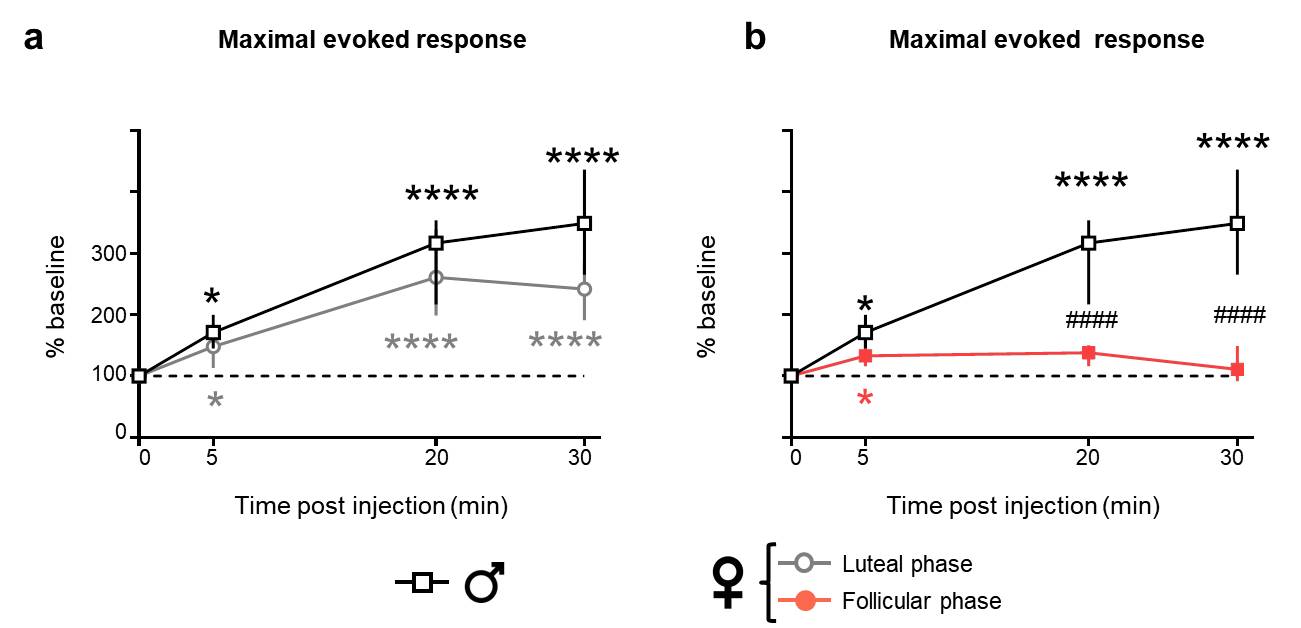
**

**Supp Figure 3: Comparison of ketamine effect in females and males**

**a)** Ketamine-induced changes of maximal evoked response in C57Bl/6J males (□, n = 67 cells / 4 mice) and females during luteal phase (■, n = 82 cells / 6 mice). **b**) Ketamine-induced changes of maximal evoked response in male WT (□, n = 67 cells/ 4 mice) and females during follicular phase (■, n= 87 cells / 5 mice).


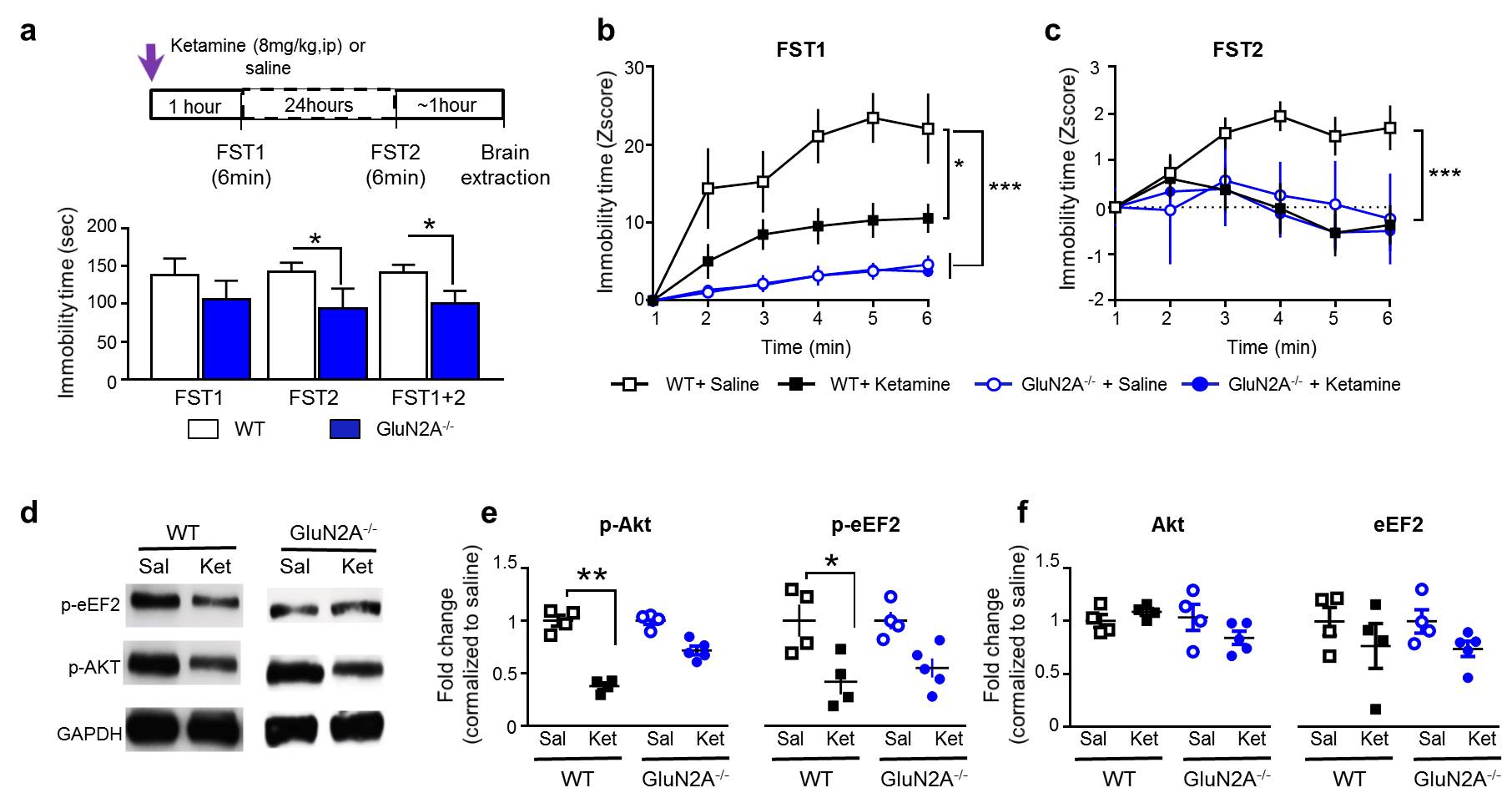


**Supp Figure 4: Absence of ketamine induced antidepressant effect in GluN2A^-/-^ mice.**

**a)** Experimental design (upper) and comparison of immobility time (lower) measured during the last 4 minutes of the forced swim test at 1 hour (FST1) and 24 hours (FST2) after injection of saline in WT and GluN2A^-/-^ mice. **b)** FST1 time course: immobility time over 6 minutes of the test normalized to the first minute. WT + saline (☐, n = 6), WT + ketamine (■, n = 6), GluN2A^-/-^ + saline (**○**, n = 6), GluN2A^-/-^ + ketamine (●, n = 7). **c**) FST2 time course: immobility time over 6 minutes of the test normalized to the first minute. WT + saline (☐, n = 9), WT + ketamine (■, n = 8), GluN2A^-/-^ + saline (**○**, n = 6), GluN2A^-/-^ + ketamine (●, n = 7). Two-way ANOVA with Turkey multiple comparison (* p < 0.05, *** p < 0.001). **d**) Sample Western blots. **e**) Quantification of p-Akt and p-eEF2 protein expression in the frontal cortex following saline or ketamine injection (n = 4 WT + saline, WT + ketamine and GluN2A^-/-^ + ketamine; n = 5 GluN2A^-/-^ + ketamine; Kruskal-Wallis with multiple comparison; p-Akt, ** p < 0.0001, * p-eEF2 p=0.0033) **f**) Quantification of Akt and eEF2 protein expression in frontal cortex of WT and GluN2A^-/-^ mice following saline or ketamine injection (Kruskal-Wallis with multiple comparison; Akt, p = 0.106, eEF2, p = 0.27).

**Materials and methods**

*In vivo* single-unit recordings: Cortical activity in the binocular zone of V1 was recorded using multichannel probes (A1x16–3 mm 50_177, Neuronexus Technologies), and the signal was amplified, thresholded and discriminated (SortClient, Plexon Technologies). Full-screen sine wave gratings (100% contrast, 0.03 cpd, 2 Hz) were presented (mean luminance = 32 cd/m^2^). Orientations varying between 0° and 360° (12 steps, 30° spacing) were presented in random order (3 s each, 3-10 repeats). The evoked response was defined as the spike response at the preferred orientation. Based on individual characteristics of well-isolated waveforms (Offline Sorter, Plexon Technologies), we selected and analyzed presumptive regular-spiking excitatory and fast-spiking inhibitory cells^34^ using SigTOOL in Matlab^77^. Criteria were imposed to exclude neurons with weak visual responses. Cells with a maximal evoked response > 0.5 spike/s and significantly above spontaneous activity (Wilcoxon signed-rank test) were kept in the analysis.

*In vitro* whole-cell recordings: Isoflurane-anesthetized PV-GFP adult mice were decapitated and their brains quickly removed into chilled, oxygenated ACSF containing (mM): 125 NaCl, 25 glucose, 25 NaHCO_3_, 2.5 KCl, 2 CaCl_2_, 1.25 NaH_2_PO_4_ and 1 MgCl_2_. Coronal slices of V1 (300 µm) were sectioned on a vibrating microtome (Leica Microsystems, VT1200S) and incubated at room temperature until placed in a recording chamber. Patch pipettes (4-6 MΩ) were pulled on a DMZ micropipette puller (Dagan Corporation).

PV-expressing cells in binocular V1 were identified by fluorescence in PV-GFP transgenic animals. Whole-cell capacitance was compensated and the initial series resistance was compensated about 60%. Data were acquired from cells with resting potential below -55 mV, an initial series resistance < 20 MΩ, and overshooting action potentials. The voltage-clamp internal solution contained (mM): 100 CsMeSO_3,_ 20 KCl, 10 HEPES, 4 Mg_2_ATP, 0.3 GTP sodium salt, 10 phosphocreatine disodium salt, 3 QX-314 (pH = 7.2 with KOH). Recordings were performed at room temperature (22-24°C) and data collected at a sampling rate of 10 kHz using an Axopatch 1D amplifier (Axon Instruments), low-pass filtered at 2 kHz, and digitized using an ITC-18 (InstruTech).

Isolated NMDA receptor-mediated excitatory post-synaptic potentials (EPSC_NMDA_) were evoked by pulses of electrical stimulation delivered to cortical layer 4 (200µs) with an ACSF-filled glass pipette and a constant current stimulus isolator (Iso-Flex, A.M.P.I.). A stimulus intensity was chosen that elicited a maximal EPSC_NMDA_ (30-100μA) which was further isolated using a cocktail of drugs to block GABA_A_ (bicuculline, 10 µM; Sigma), GABA_B_ (SCH-50911, 10 µM; Tocris Bioscience), AMPA (CNQX, 20 µM; Tocris Bioscience), and muscarinic acetylcholine (atropine, 1 µM; Sigma) receptors. The effects of ketamine (10 µM; Hospira Inc) on EPSC_NMDA_ amplitudes were evaluated 15 minutes after bath application. The NMDA receptor-mediated component was ultimately verified using specific antagonists (CPP, 20 µm; AP-5, 50µM; Tocris Bioscience). Custom-designed IGOR (WaveMetrics) programs were used for data acquisition and analysis.

Identification of the mouse estrous cycle: We performed vaginal cytology as defined previously^33^. Stages of the estrous cycle were determined by observing the presence of leukocytes, cornified epithelial cells, and nucleated epithelial cells in the fluid. In the estrus state, cornified epithelial cells were predominant, while in metestrus, a large number of leukocytes and nucleated cells were observed. In the diestrus stage, there were few leukocytes, nucleated or cornified epithelial cells.

Bootstrapping Analysis: Data were re-sampled 1000 times using the bootstrap/bootfun function in MATLAB to find new bootstrapped medians at each time point. Ranges shown are 95% confidence intervals of medians.

Forced Swim Test (FST): C57BL/6J and GluN2A^-/-^ males (4-6 months old) were tested in the FST at 1h and 24h after ketamine (8mg/kg, i.p.) or saline (i.p.) injection. They were placed in a transparent Plexiglass beaker (height: 25 cm, diameter: 15 cm) containing 3000ml of clear water at 24-26°C for 6 minutes. Mice were not able to touch the bottom of the beaker with their tail. The tests were performed under dim light conditions (~30LX). To avoid subjective evaluation, sessions were recorded by a digital video camera and automated quantification of mouse behavior was performed offline. Using ImageJ and Matlab, we evaluated immobility time based on Motion Energy Analysis^78, 79^. Temporary changes in grayscale distribution were quantified over 6 minutes of the test; a threshold at 60% of maximum changes was used to identify movements. Repeated FST disrupted behavior by increasing immobility time during the first minute of testing on the second day in comparison to the first test for both strains injected with saline (3 ± 0.6 vs 19 ± 3.3 sec in WT, p<0.01; 8 ± 1.9 sec vs 22 ± 3.2 sec in GluN2A^-/-^, p <0.01). To control for any learning process, we therefore quantified immobility time increase normalized to the first minute of each test.

Western Blot: Mice were briefly anesthetized with 3% isoflurane and oxygen. Cervical dislocation was performed and the brain quickly removed and rinsed in sterile saline. The frontal part of the cortex was dissected, frozen in liquid nitrogen and stored at -80°C until RNA or protein extraction. Cortices were homogenized by sonication in RIPANaPO4 buffer. Total protein concentrations were determined by micro BCA methods (Thermo Scientific) and optical density measurement with ImageJ software. Protein samples (40 µg) were loaded in individual wells and separated by 10% SDS/PAGE and transferred to nitrocellulose membranes (Whatman). The following primary antibodies were used: rabbit monoclonal anti-Akt (1:1000, Cell Signaling Technology, # 4691S), rabbit monoclonal anti-phospho Akt (Ser473) (1:1000, Cell Signaling Technology, #4060S), rabbit anti-eEF2 (1:1000, Cell Signaling Technology, #2332S), rabbit anti-phospho eEF2(T56) (1:1000, Cell Signaling Technology, #2331S), and mouse monoclonal anti-GAPDH (1:40000, Abcam, ab8245). Blots were developed to film after ECL treatment with KwikQuant Imager (Kindle Biosciences LLC). Quantifications were normalized to GAPDH levels for each sample.

References

77 Lidierth M. sigTOOL: A MATLAB-based environment for sharing laboratory-developed software to analyze biological signals. *J Neurosci Methods* 2009; **178**: 188–96.

78 Grammer K, Honda M, Juette A, Schmitt A. Fuzziness of nonverbal courtship communication unblurred by motion energy detection. *J Pers Soc Psychol* 1999. doi:10.1037/0022-3514.77.3.487

79 Kupper Z, Ramseyer F, Hoffmann H, Kalbermatten S, Tschacher W. Video-based quantification of body movement during social interaction indicates the severity of negative symptoms in patients with schizophrenia. *Schizophr Res* 2010. doi:10.1016/j.schres.2010.03.032
